# Self-Powered Pressure Sensor with fully encapsulated 3D printed wavy substrate and highly-aligned piezoelectric fibers array

**DOI:** 10.1038/s41598-017-07360-z

**Published:** 2017-07-28

**Authors:** Yiin Kuen Fuh, Bo Sheng Wang, Chen-Yu Tsai

**Affiliations:** 1 Department of Mechanical Engineering, National Central University, Taoyuan City, Taiwan; 2 Institute of Energy Engineering, National Central University, Taoyuan City, Taiwan

## Abstract

Near-field electrospinning (NFES) is capable of precisely deposit one-dimensional (1D) or two-dimensional (2D) highly aligned micro/nano fibers (NMFs) by electrically discharged a polymer solution. In this paper, a new integration of three-dimensional (3D) architectures of NFES electrospun polyvinylidene fluoride (PVDF) NMFs with the 3D printed topologically tailored substrate are demonstrated in a direct-write and *in-situ* poled manner, called wavy- substrate self-powered sensors (WSS). The fabrication steps are composed of the additive manufacture of 3D printed flexible and sinusoidal wavy substrate, metallization and NFES electrospun fibers in the 3D topology. This 3D architecture is capable of greatly enhancing the piezoelectric output. Finally, the proposed piezoelectrically integrated 3D architecture is applied to the self-powered sensors such as foot pressure measurement, human motion monitoring and finger-induced power generation. The proposed technique demonstrates the advancement of existing electrospinning technologies in constructing 3D structures and several promising applications for biomedical and wearable electronics.

## Introduction

Self-power system is on the huge demand for portable or wearable electronics for the ubiquitous computing systems. One of the key underlying principles is the piezoelectricity. Piezoelectric materials such as Lead Zirconate Titanate (PZT), zinc oxide (ZnO) and polyvinylidene fluoride (PVDF) provide a feasible way to effectively harvest energy from ambient sources or human actions rather than depending on cell batteries^1–4^. For the operating mechanism of self-powered nanowires (NWs) sensors and the development of biocompatible and biodegradable devices, a highly focus area has been attracted great attention on recent years^5–7^. The nano-related materials of piezoelectric properties have been studied broadly since 2006, the first piezoelectric energy harvester constructed by ZnO NWs arrays^8^ had been developed as a promising and new power sources and tremendously increased investigations on self-powered devices were embarked on refs 9–13. For the fabrication of nano fibers, the old and widely adopted technique is called electrospinning. The fundamental principle of electrospinning is electrically driving the polymeric jet from the Taylor cones of premixed solution. A rich diversification of materials includes ceramics, polymers, and composites can be deposited with diameter ranging from tens of nanometers to few microns. The inevitable limitation of conventional electrospinning process is strongly beset by the geometric and morphological control^14^. The recent study presented a controllable, direct-write and patterning manners via near-field electrospinning (NFES) which used PVDF as main piezoelectric material^15–17^. In particular, a popular piezoelectric polymer of PVDF has been studied widely due to its highly stretchable flexibility, biocompatibility, and cheap expense^18–20^. Consequently, electrospinning technique is comparatively simple, economical and versatile process to fabricate nano/micro fibers (NMFs) based piezoelectric nanogenerator (NG)^21–24^ from polymers or composites materials. Other principles of energy harvesting based on the mechanical interaction are rendering to the phenomena of triboelectricity and electrostaticity. Several demonstrated generators for effectively converting mechanical energy to electricity ranging from piezoelectric NGs, triboelectric nanogenerators (TENGs), electret generators, and electrostatic generators^25–28^. For the actual demonstration of self-powered devices, a functional tactile and highly sensitive flexible pressure sensor with microstructured rubber dielectric layers were demonstrated^29^. Similar device was also fabricated with organic transistors or micro-structured rubber layer for the application of tunable and flexible pressure sensor arrays have been reported^30^. A newly self-powered triboelectric pressure sensor based on hemispheres-array-structure was reported to firmly build in enclosed and packaged system without any spacer^31^. The advancement of NGs provides us with an alternate energy paradigm shift and pushes forward the investigation on batteryless, wireless self-powered systems^32, 33^. On the other hand, previous reports on the combination of electrpspun nano- and imprinted micro-scale patterns can also offer as the topographic cues to the engineered vascular application^34^. While the additive manufacturing method of 3D printing can also be used as a promising tool for the production of electrospun scaffolds with tailored three-dimensional micro-patterns^35^. In this paper, the 3D architectures of NFES electrospun PVDF NMFs are functionally integrated with the 3D printed topologically tailored substrate. The fabricated WSS is capable of greatly enhancing the piezoelectric output and directly applying to the foot pressure measurement and human motion monitoring in a self-powered fashion.

## Experimental Results

**Figure 1:**
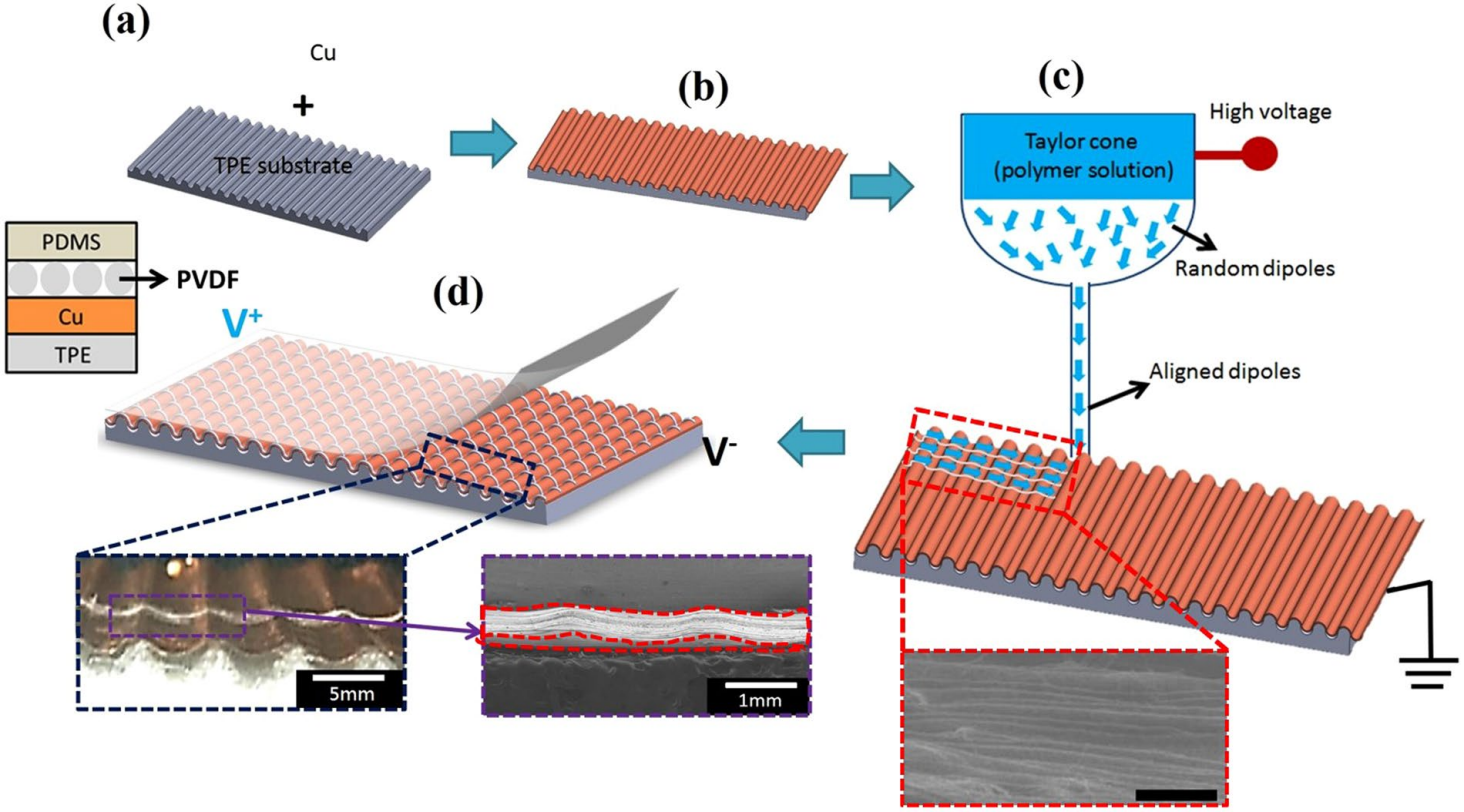
(**a**) Schematic of the near-field electrospinning process to fabricate direct-write PVDF fibers on top of wavy substrate. (**b**) Mechanical adhesion of wavy substrate with Cu foil as conductive electrode. (**c**) Electrospun PVDF fibers on top of Cu-adhesive wavy substrate via NFES. (**d**) Fully encapsulation with PDMS (bottom inset: optical photo and SEM image of PVDF fibers deposited on top of wavy substrate, exhibiting the wavy and 3D topology). Scale bar is 200 μm.

The piezoelectric generator with fibers of wavy and three-dimensional (3D) topology has been demonstrated in this article and the fabrication process consists of four main steps can be illustrated in Fig. 1. Initially the Cu foil was mechanically attached to the thermoplastic elastomer (TPE) substrate with 3D printed wavy surface (Fig. 1a, TPE substrate thickness is about 2 mm). Figure 1b shows the adhesively bonded wavy substrate and the PVDF piezoelectric fibers were continuously deposited on the Cu foil electrode via *in-situ* poled NFES technique (Fig. 1c, NFES processing setup is the followings: needle top to Cu collector distance ~1.5 mm, the electrospinning process parameters used in this case are 16 wt% PVDF, solvent (Dimethylformamide DMF: acetone with 1:1 weight ratio), 4 wt% fluoro-surfactant (Capstone FS-66)). NFES has the great controllability to deposit very delicate pattern of fibers into the substrate. The final packaging step is utilized PDMS to fully encapsulate and isolate from the environmental disturbances. Detail information regarding the experimental procedure used to make the 3D printed topologically tailored substrates and the NFES electrospun NMFs are given in the Supplementary Information Table S1. The inset in Fig. 1d shows PVDF fibers deposited on top of wavy substrate, exhibiting the wavy and 3D topology in scanning electron microscopy (SEM) image of the fabricated fibers. Based on the morphological SEM of the electrospun fibers (inset of Fig. 1c), diameter range of NMFs are found to be between 1 and 5 μm, length can be continuously deposited as long as ~35 mm the fiber pattern have the same periodicity of ~15 μm. However, please noted that the NFES is performed at atmospheric condition (without vacuum), therefore, the environmental disturbance will inevitably result in the accumulation of electrospun NMFs in some regions, as indicated in SEM. Nonetheless, the highly-aligned piezoelectric fibers array can be observed. The novelty of this research is to physically demonstrate the topologically 3D wavy structures with enhanced piezoelectric and electrical performance. Due to the spinnability of PVDF solution, the diameters of as-spun PVDF fibers might range from hundreds nm to several μm^36^. The continuous deposition of PVDF NMFs was fabricated under restricted operating region at the sacrifice of diameter variation of NMFs which was identified in previous research^37^. In order to validate the evidence of the aligned dipoles and related piezoelectric property, we collected the spectroscopic evidence of Fourier transform infrared spectroscopy (FTIR) and X-ray diffraction (XRD) in Supplementary Information Figures S1 and S2 respectively. Piezoelectricity of the electrospun NMFs can be majorly validated by the strong presence of β-phase peaks, as indicated in the FTIR and XRD results of NFES PVDF fibers.

**Figure 2:**
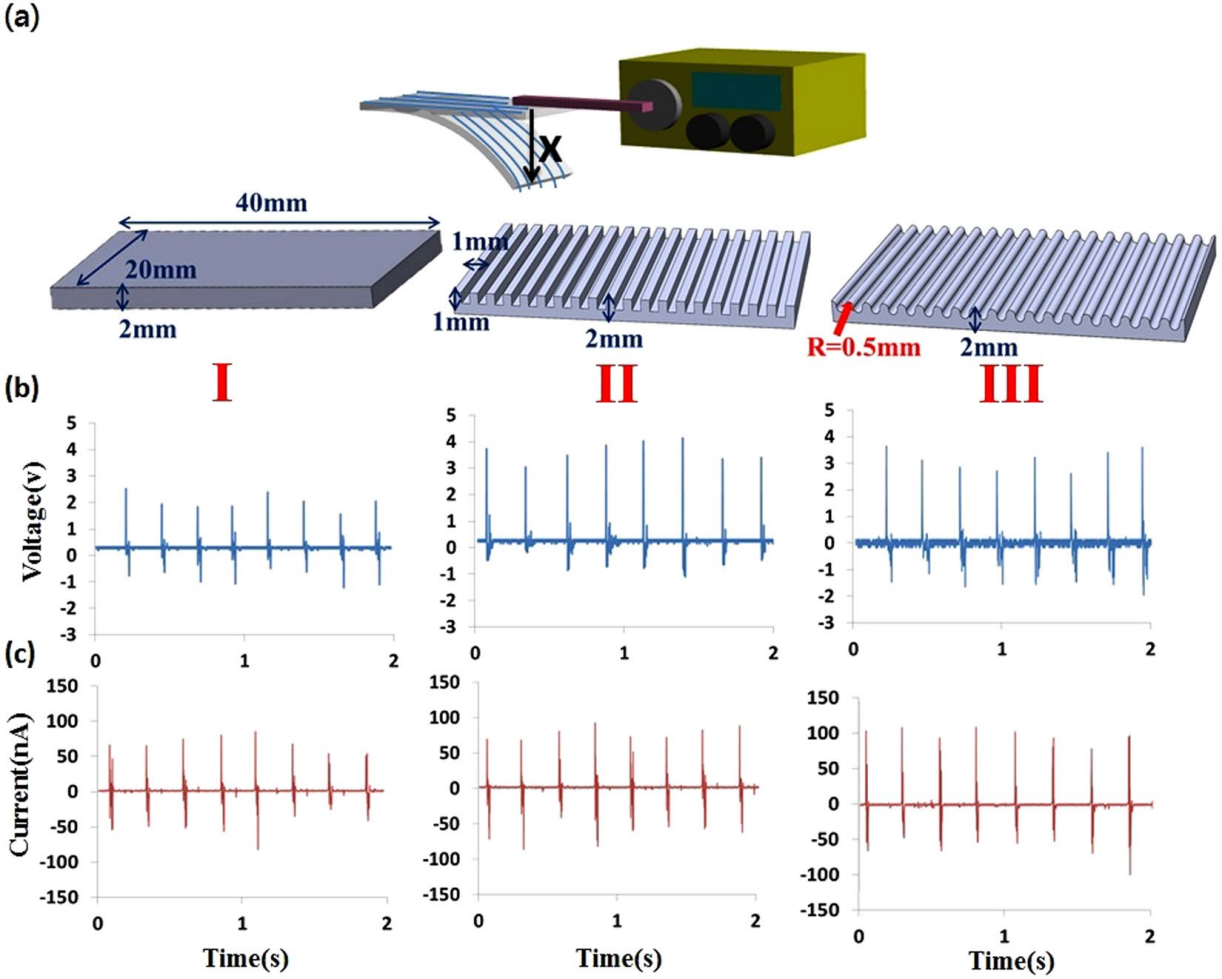
(**a**) Schematic of the experimental setup and three topologically different surfaces were investigates as: planar surface (I), square surface (II) and sinusoidal surface (III). (**b**) Output voltage and (**c**) current versus stretching-releasing deformation strain of 0.5% at 4 hz. The topologically tailored devices (I/II/III) can stably produce about average output voltage 2 V/3.5 V/4 V and output current 50 nA/70 nA/100 nA, respectively during the cyclic deformation.

We produced three different devices with purposely tailored configurations, which have different topologies as illustrated in Fig. 2a and denoted as follows: planar surface (I), square surface (II) and sinusoidal surface (III). We name the fabricated devices as wavy-based-substrate self-powered sensors (WSS**)**. All samples have a dimension of 40 mm × 20 mm × 2 mm. For square wave surface, the height and pitch of square wave arrays are 1mm. For the Sinusoidal surface, the height/pitch is 1mm while the top radius is 0.5mm. For the experimental setup to measure the output voltage and current versus cyclic stretching-releasing deformation, a commercially available DC motor (RS-545SH) was utilized and experimentally operated at a strain of 0.5% and 4 hz. Voltage and current measurements were performed using an oscilloscope (Agilent DSO1014A) and an electrochemical analyzer (model CHI611E). In Fig. 2b, the three different WSS devices (planar, square and sinusoidal surfaces) with a total of 600 deposited PVDF fibers can generate output voltage about 2 V, 3.5 V and 3.5 V, respectively. Figure 2c indicates that the output current signals of the different surfaces measured as about 50 nA, 70 nA and 100 nA, respectively. In addition, the stability of the WSS was an essential factor to ensure its follow-up applications. Three days of continuous output voltage and output current of the WSS operating at 4 hz were given in detail in Supplementary Information Figure S3. From the experimental results, the device exhibits robust stability and only a small variation of peak output voltage is measured, indicating the highly stable power generation of the WSS. To be subsequently characterized as a self-powered sensor via the piezoelectric principle, the validated polarity of forward and reverse connections measurements were also performed in Supplementary Information Figure S4. It is crucial to the piezoelectric generator to validate the polarity test such that the background or triboelectric signals can be excluded and authentic piezoelectric responses can be confirmed. Moreover, the superposition principle of voltage signals was also validated by the serially connected of two and three WSSs, as presented in Supplementary Information Figure S5. Another experiment was also performed to exclude the triboelectric effect by using the fully-encapsulated, non-piezoelectric fibers (PVP/ZrO2) and fibers-free substrates subject to continuous stretch and release as presented in Supplementary Information Figure S6. Both experiments further confirm that the electrical outputs of developed WSSs are genuinely the piezoelectric signals.

**Figure 3:**
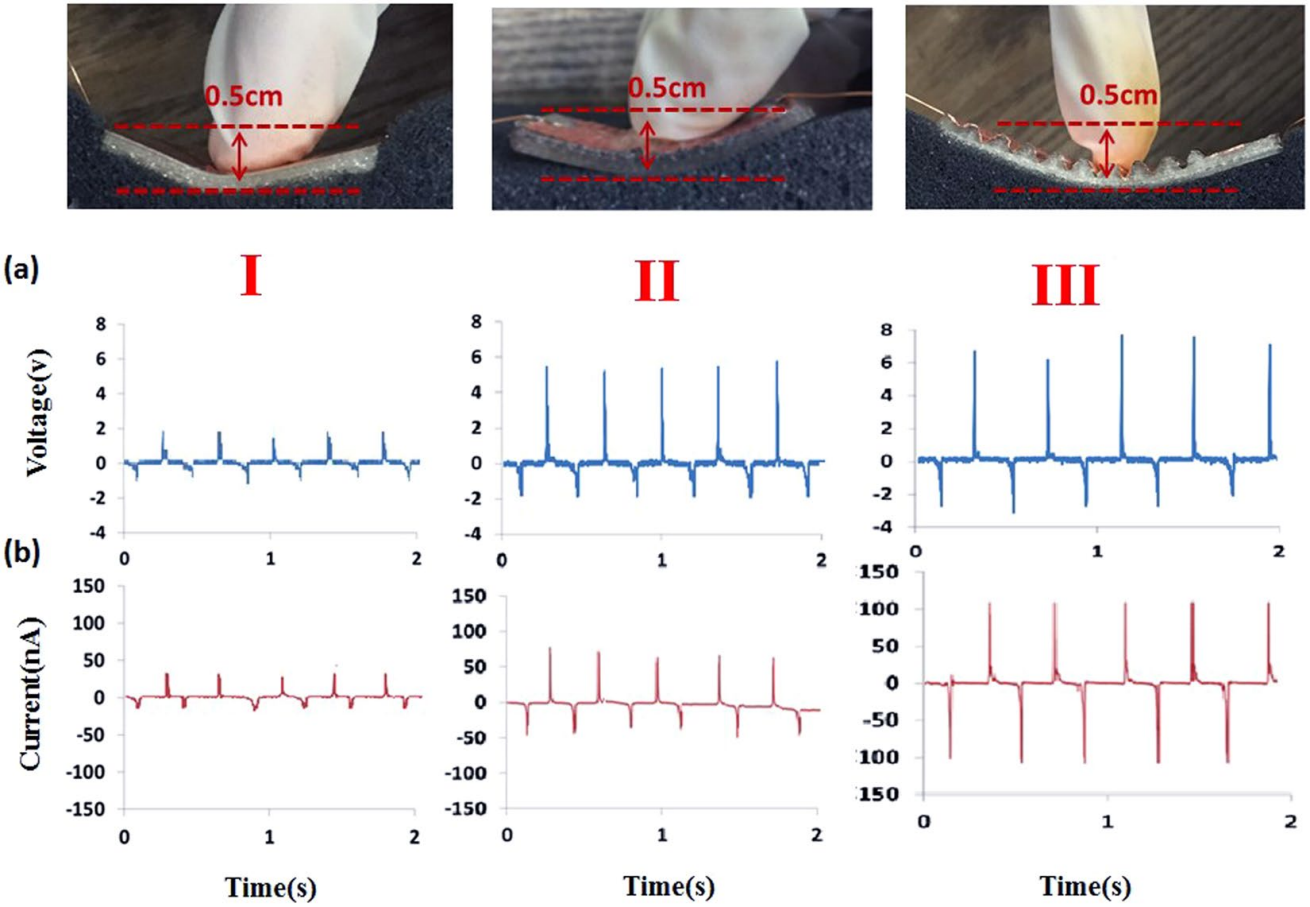
Measured output voltage and current. Placed the device on the cotton fabric and performed the press/release cycles repeatedly on three different devices to obtain open-circuit (**a**) voltage and (**b**) current. The I/II/III devices produces average output voltage 2 V/5 V/7 V and output current 50 nA/80 nA/110 nA, respectively.

Three different devices undertaken the tests of repeatedly press/release cycles are denoted as follows: planar surface (I), square surface (II) and sinusoidal surface (III). In Fig. 3 The WSS was settled on the cotton fabric and pressed by a finger, in Fig. 3a the WSS generated power output voltage was measured at an average of 2 V/5 V/7 V when three different devices were against finger-induced strain (the center displacement is measured about 0.5 cm for WSS). Similarly, the same test were performed to measure the current output performance as a comparison with Fig. 3b, the WSS generated power output current was measured at an average of 50 nA/80 nA/110 nA, respectively. The results showed that the device III with sinusoidal wavy surface had the best output and the reasons may be primarily attributed the longest fiber length as electrospun by NFES process. Overall, the three tested device shared the resemblance of good stability and small variation of peak output voltage, indicating the relatively stable power output as the sustainable sensors application. In addition, finite element simulation (DEFORM) was performed for the experienced strain states of topologically different substrates and presented in the Supplementary Information Figure S7. The simulation strain values indicated that the sinusoidal wavy surface had the larger strain exerted on the substrate and thus, the better electrical output.

**Figure 4:**
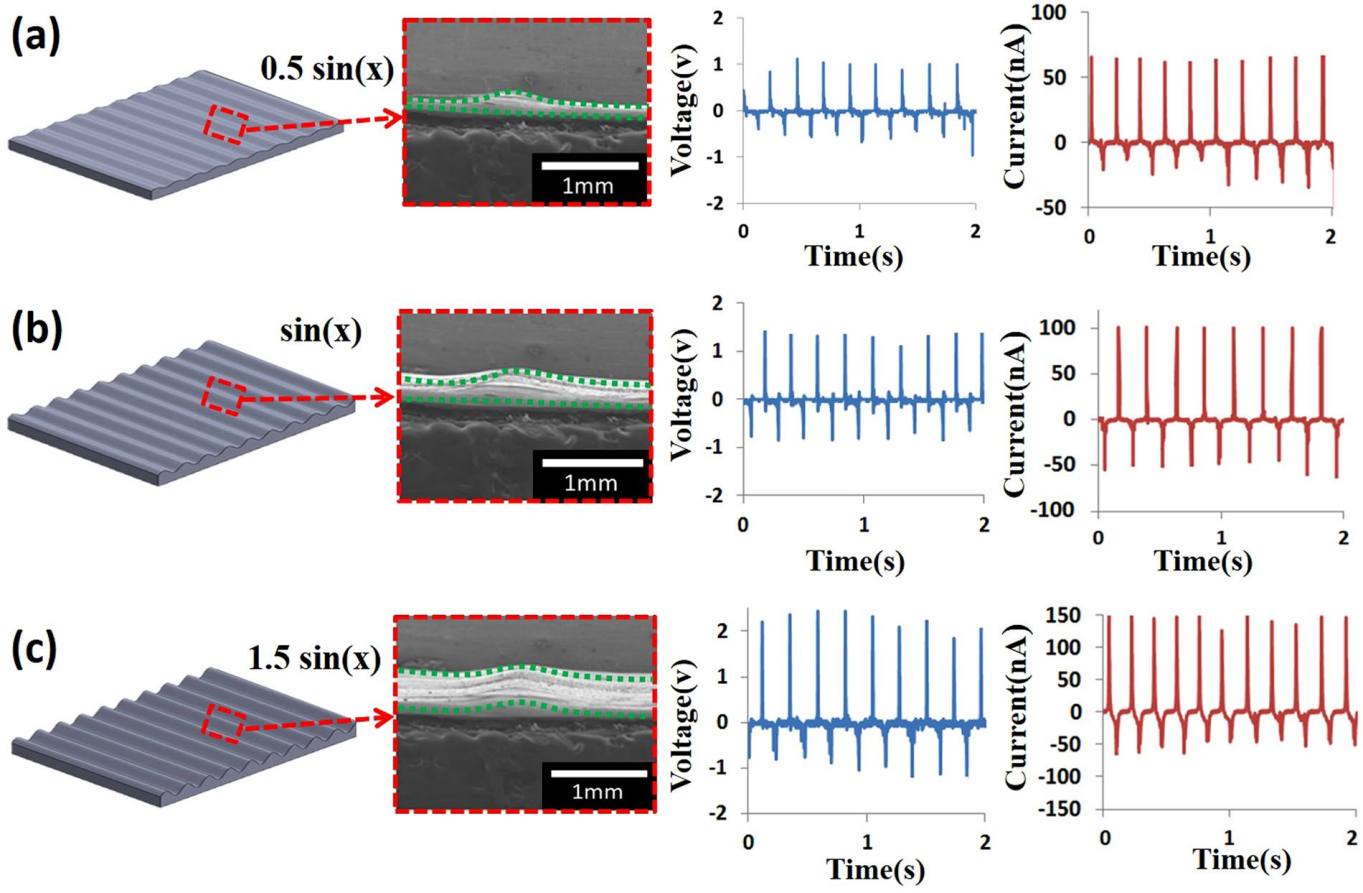
Effect of sinusoidal amplitude (Asin(x)) for wavy surfaces and corresponding output voltage and current. For the amplitude A is chosen as (**a**) 0.5 sin(x), (**b**) sin(x), (**c**) 1.5 sin(x). All dimensions are in mm.

From the experimental results Fig. 3 showed that the device of sinusoidal surface had the best output, the effect of amplitude height of the purposely tailored wavy configurations is further investigated as illustrated in Fig. 4a–c. The investigated amplitude heights for the sinusoidal surface are 0.5, 1 and 1.5 mm respectively. The total height of the device is fixed at 2 mm. Place the devices on the cotton fabric and cyclically perform the press/release operations with a displacement of 0.5 cm to obtain open-circuit voltage and short-circuit current. The experimental results in Fig. 4a–c show that the average output voltages and currents for the amplitude of 0.5 mm/1 mm/1.5 mm are 1 V/1.5 V/2 V and 60 nA/100 nA/150 nA respectively. For the actual application of above-mentioned device, we further integrate the wavy substrate with the button of the mobile phone. First, three different amplitudes of sinusoidal wavy surfaces of A sin(x), where the amplitude A values are 0.5,1, 1.5 mm respectively, are combining with the same polymeric substrates as illustrated in Supplementary Information Figure S8a. The average output voltage is experimentally found to be proportional to the amplitude of wavy amplitude and the reason is mainly attributed to longer electrospun fibers and higher strain experienced during the repeated press/release cycle. Supplementary Information Figure S8b shows the application of the integrated WSS device with the button of mobile phone. The average output voltages and currents generated by integrated WSS device with amplitude of 0.5/1/1.5 mm height are ~1.5 V/5 V/7 V and ~5 nA/30 nA/50 nA respectively in one striking motion of button movement. This site addressable capability on the same substrate is useful for potential wearable device and smart button applications such that the button-hitting operation can be functionally discernable to any specific remote control features.

**Figure 5:**
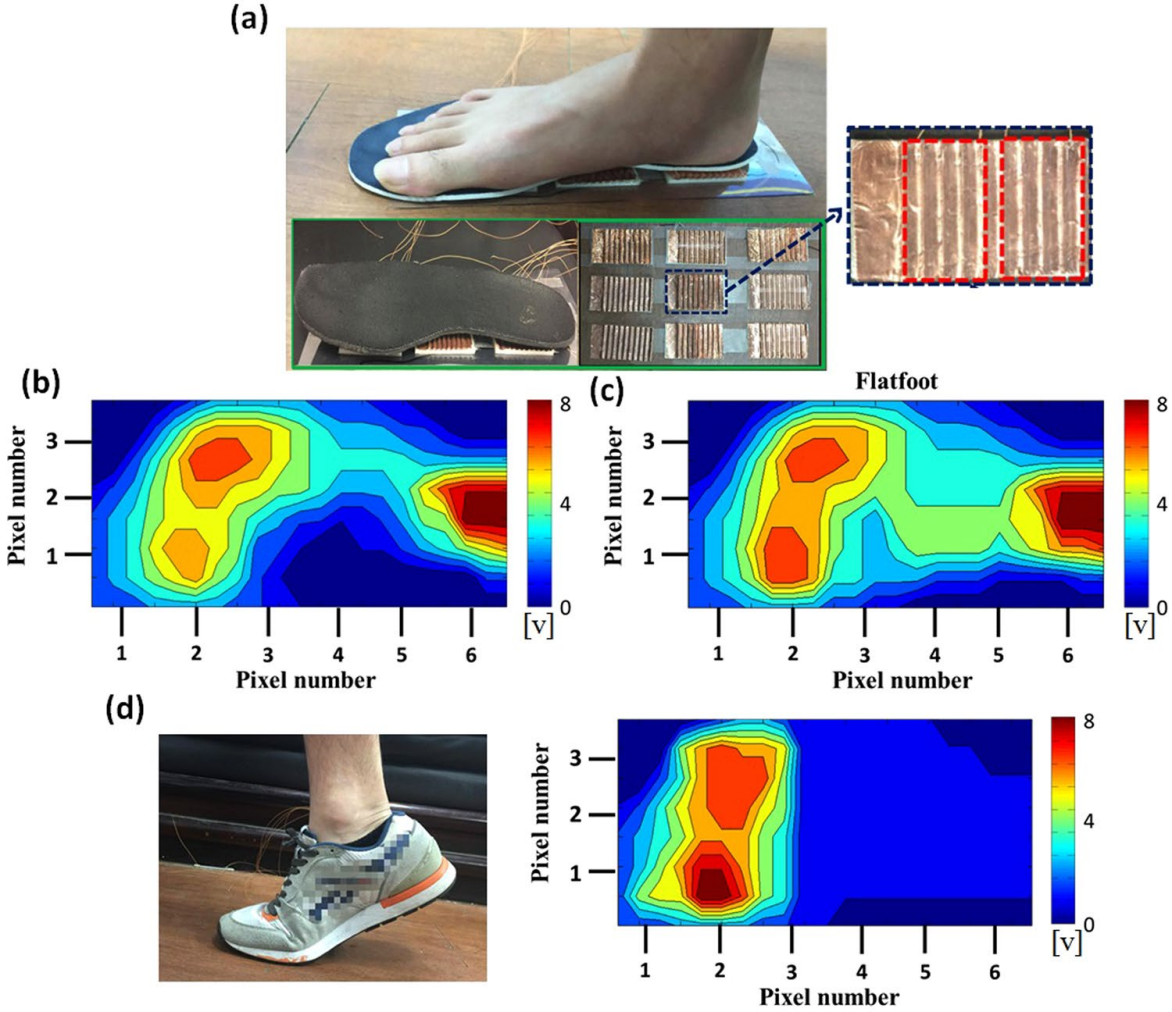
(**a**) Actual demonstration of WSS integrated self-powered foot pressure sensor arrays (3 × 3; 4.5 cm × 3 cm of dimension for each unit cell) for detecting the foot pressure (inset: visually illustrated insole on top of the foot pressure sensor device). (**b**) and (**c**) shows two dimensional contour plot mapping of pressure potential from the objects of (**b**) normal foot, (**c**) flat foot. (**d**) In a tip-toed position and the related two dimensional contours plot mapping of pressure potential.

We further demonstrated the integration of WSS as the self-powered foot pressure mapping sensor for detecting applied multiple forces in real-time. As shown in Fig. 5a, the WSS with an active area of 4.5 cm × 3 cm were arrayed on matrix patterned electrodes and the different pressures from a foot can be directly corresponded to the distribution of strain as measured by WSS. Figure 5b–d showed a 2D contour plotted for the normal foot, flat foot and tip-toed position respectively. The 2D contour plots are reconstructed based on the measured signals of each WSS array and the overall potential can be mathematically mapping to each individual WSS array. The corresponding pressure intensities to each unit array are carefully calibrated and characterized with experimental results. The measured raw output data set is given in Supplementary Information Figure S9. Furthermore, Supplementary Information Figure S10 characterized the relationship between the applied pressure and electrical voltage of the human motion-induced deformation, showing the promising potential of developed device as the self-powered pressure sensor. Theoretically and experimentally for the forces and pressure derived from human motions, the proposed WSS based pressure sensor demonstrates the potential to apply on health care monitoring system can be discernably detectable to collect pressure information, as indicated in Fig. 5b–d such that the different objects with normal, flat foot and tip-toed conditions can be clearly identified. Owing to the structurally durable and flexible design, the related symptoms of repeatedly applied forces in human motions can be fully integrated the WSS device in a self-powered manner.

## Conclusion

In conclusion, the WSS and 3D architectures of NFES electrospun PVDF NMFs is demonstrated in a direct-write and *in-situ* poled manner. Additive manufacture of 3D printed technique is also applied to fabricate the sinusoidal wavy substrate and integrated with NFES electrospun fibers in the 3D topology. This proposed 3D architecture is capable of greatly enhancing the piezoelectric output and the reasons may be primarily attributed the longest fiber length as electrospun by NFES process. Furthermore, the site addressable capability of piezoelectrically integrated 3D architecture is directly applied to the wearable device and smart button applications such that the button-hitting operation can be functionally discernable to any specific remote control features in a self-powered manner. The proposed technique has the potential to advance the existing electrospinning technologies in constructing 3D structures for biomedical and wearable electronics.

### Ethics approval and consent to participate

All authors agreed on the ethics approval and consent to participate.

## Electronic supplementary material

Supplementary information
