# Supplementary material for: Self-Powered Pressure Sensor with fully encapsulated 3D printed wavy substrate and highly-aligned piezoelectric fibers array
Source: Sci Rep. 2017 Jul 28;7:6759. doi: 10.1038/s41598-017-07360-z (PMC5533785; doi:10.1038/s41598-017-07360-z)
Supplement: Supplementary file 1 — Supplementary information [file 41598_2017_7360_MOESM1_ESM.pdf]

Self-Powered Pressure Sensor with fully encapsulated 3D printed wavy substrate and highly-aligned piezoelectric fibers array

Yiin Kuen Fuh[1,2], Bo Sheng Wang[1],Chen-Yu Tsai[1]

[1]Department of Mechanical Engineering, National Central University, Taoyuan City, Taiwan.

[2]Institute of Energy Engineering, National Central University, Taoyuan City, Taiwan.

Corresponding author: Dr. Yiin-Kuen Fuh

Mailing Address: Department of Mechanical Engineering, National Central University, No.300,

Jhongda Rd., Jhongli District, Taoyuan City 32001, Taiwan (R.O.C.)

Telephones: +886- 03-4267305 (office)

Fax: +886- 03-4254501

E-mail: michaelfuh@gmail.com

Supplementary information

3D printed TPE substrate		Near field electrospun nano/micro fibers	
Dimension	40×20×2mm	Applied voltage	1500V
Nozzle size	0.4mm	Stage speed	6cm/s
Nozzle amount	1	Needle diameter	0.17mm
Nozzle heat	230℃	Tip-to-substrate distance	1.5mm
Filament diameter	1.75mm	Syringe pump feed speed	8×10^{-5} ml/s
Point accurate	xy resolution 0.01mm z resolution 0.00125mm	Electric field	$\sim10^{6}$ V/m

Table S1 Detail specifications of 3d printed TPE substrate and NFES electrospun NMFs

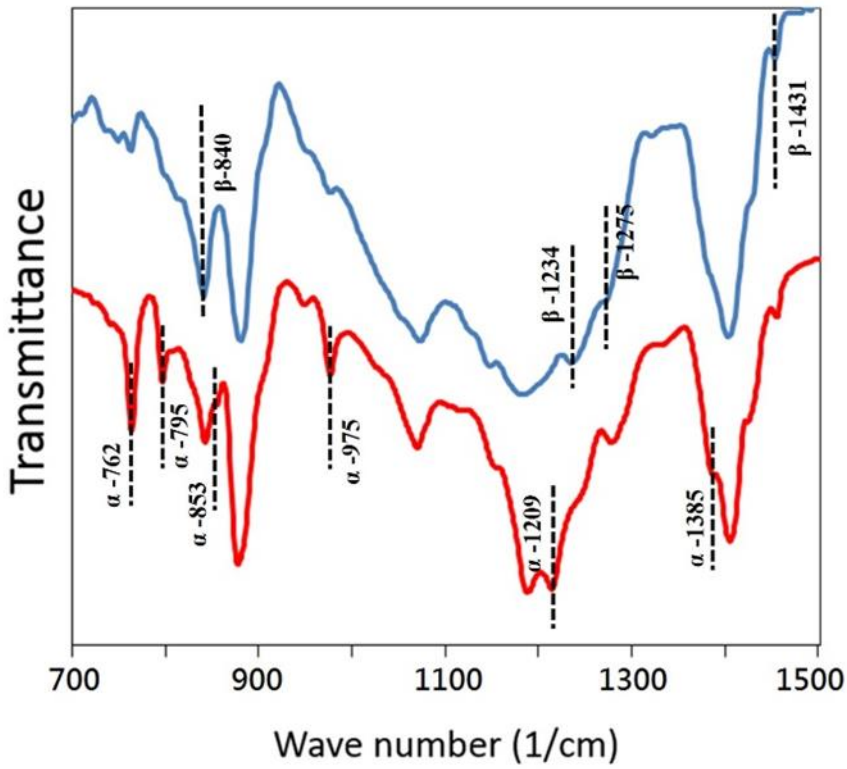

Fig. S1 FTIR spectra of the PVDF powder and electrospinning PVDF fibers. The characteristic transmittance bands of the β-phase are measured at 847, 1240, 1278, and 1435 cm^{-1} in PVDF fiber (blue line). On the other hand, the characteristic transmittance bands of the non-polar α-phase were observed in PVDF powder (red line) at 760, 795, 855, 982, 1218, and 1392 cm^{-1}.

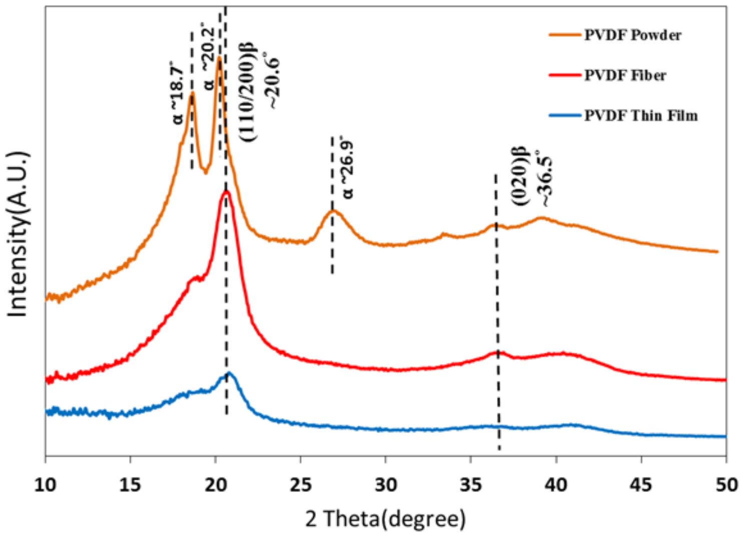

Fig. S2 XRD patterns of original PVDF powder (orange line), NFES PVDF fiber (red line) and conventional electrospinning PVDF thin film (blue line). XRD measurement results show two obvious diffraction peaks at 2θ = 18.7°, 20.2° and a weaker peak at 2θ = 26.9° corresponding to 020, 110 and 021 reflections of the α-phase (monoclinic) crystal, respectively. The NFES PVDF fiber (red line) and conventional electrospinning PVDF thin film (blue line) are both observed a very strong diffraction peak at 2θ = 20.6° and a weak peak at 2θ = 36.5° corresponding to 110/200 and 020 reflections of the β-phase (orthorhombic) crystal, respectively.

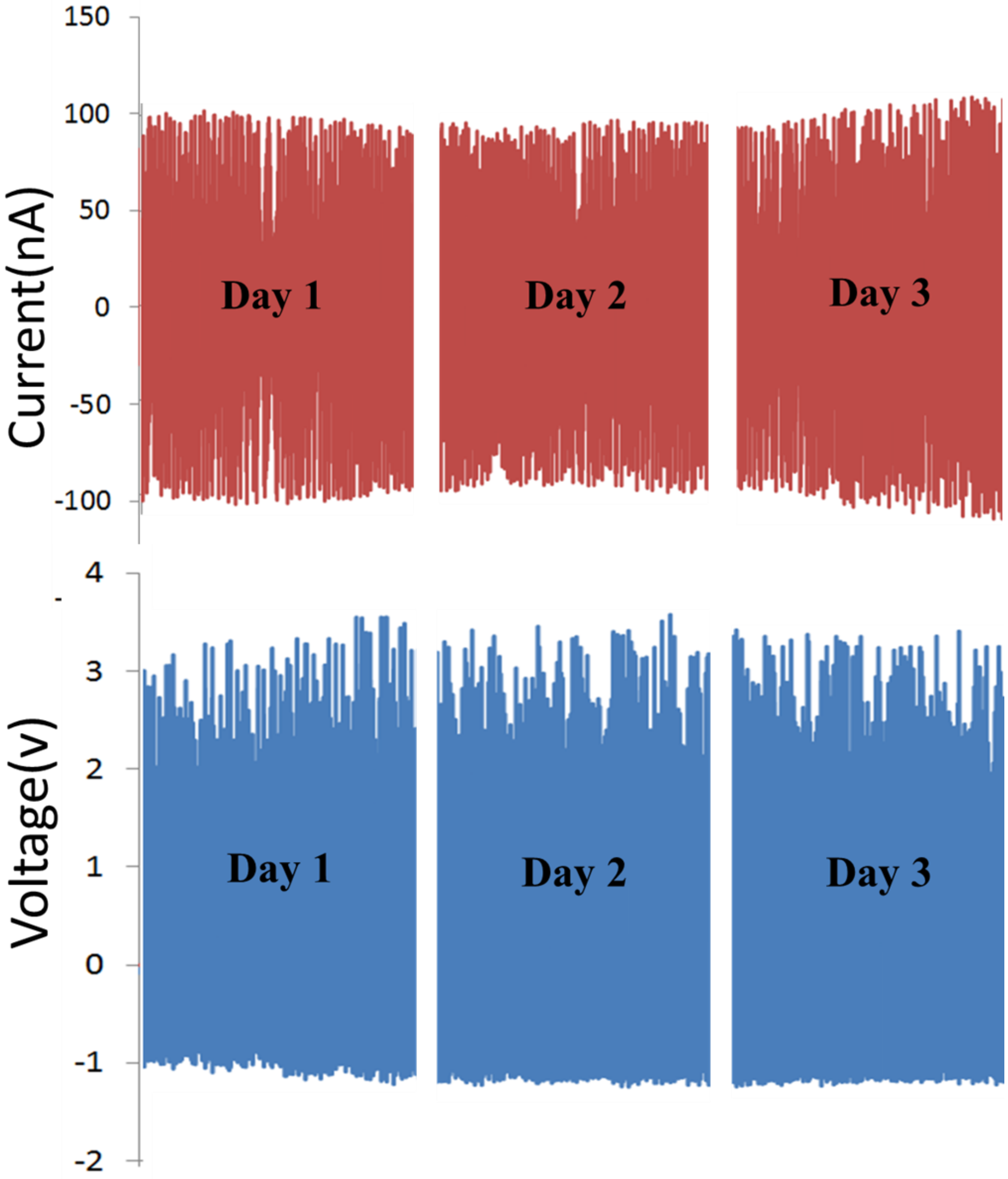

Figure S3. Stability tested for five consecutive days. The output (a) voltages and (b) currents of the WSS operating at strain of 0.5% 4Hz for 10 minutes per day.

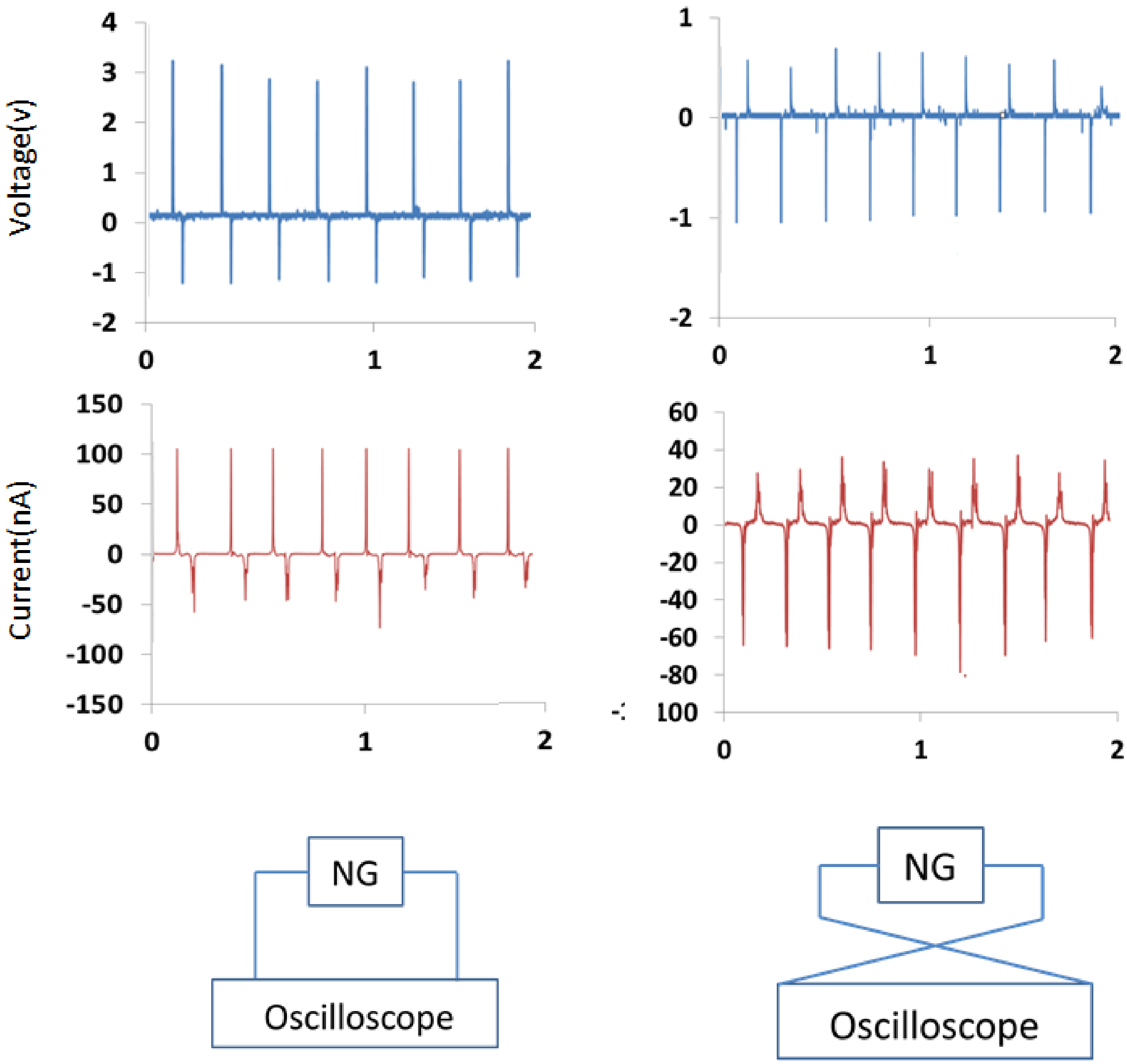

Figure S4. Validated tests of polarity as implemented via forward and reverse connections measurements. The shape of output signal changed as switching the measurement polarity. (a) The peak voltage and currents generated by the WSS of about 4V and current of about 100nA were obtained in the forward connection. (b) The output voltages and currents generated by the WSS of about 1V and about 50nA in the reverse connection. All the tests were operating at strain of 0.5% and 4Hz.

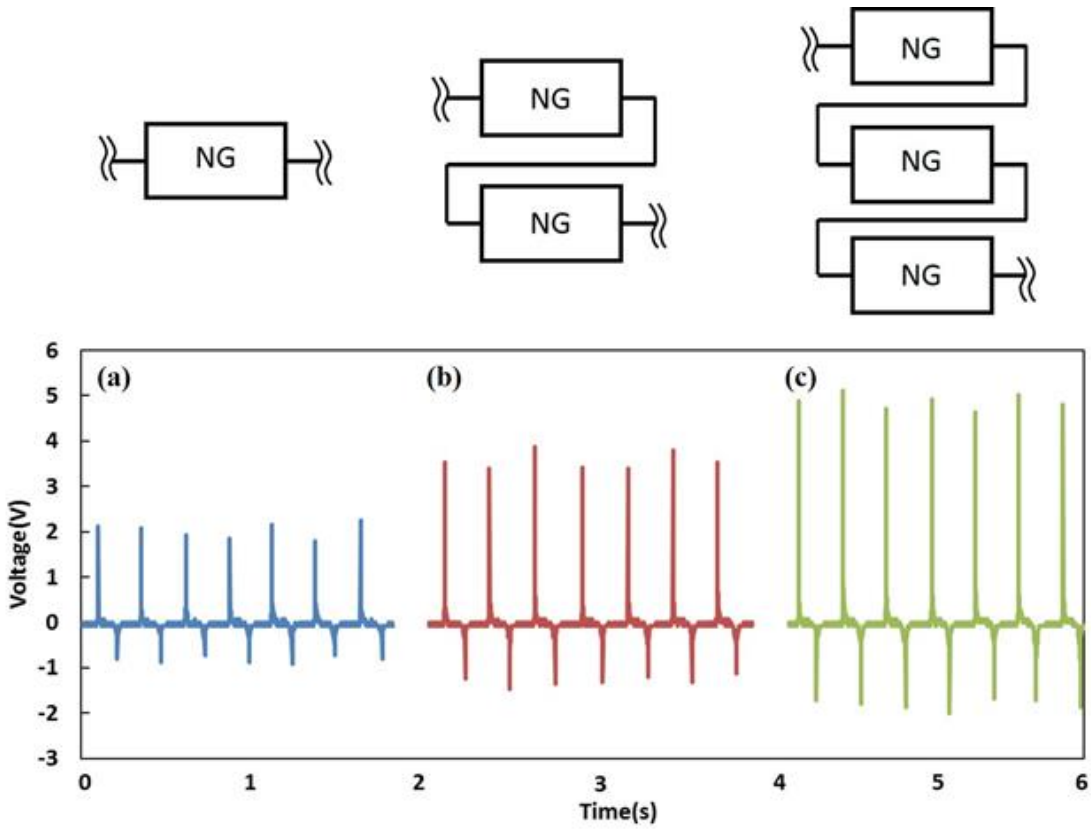

Figure S5. Output voltages of (a) WSS #1, (b) WSS #2 and (c) WSS #3 subject to continuous stretch and release. Output voltages constructively add when (d) two WSSs (WSS #1+ WSS #2) (e) three WSSs (WSS #1+ WSS #2+ WSS #3) are in serial connection. All data are measured when the two WSSs are operated at the same strain (0.5%), strain rate, and frequency (4Hz).

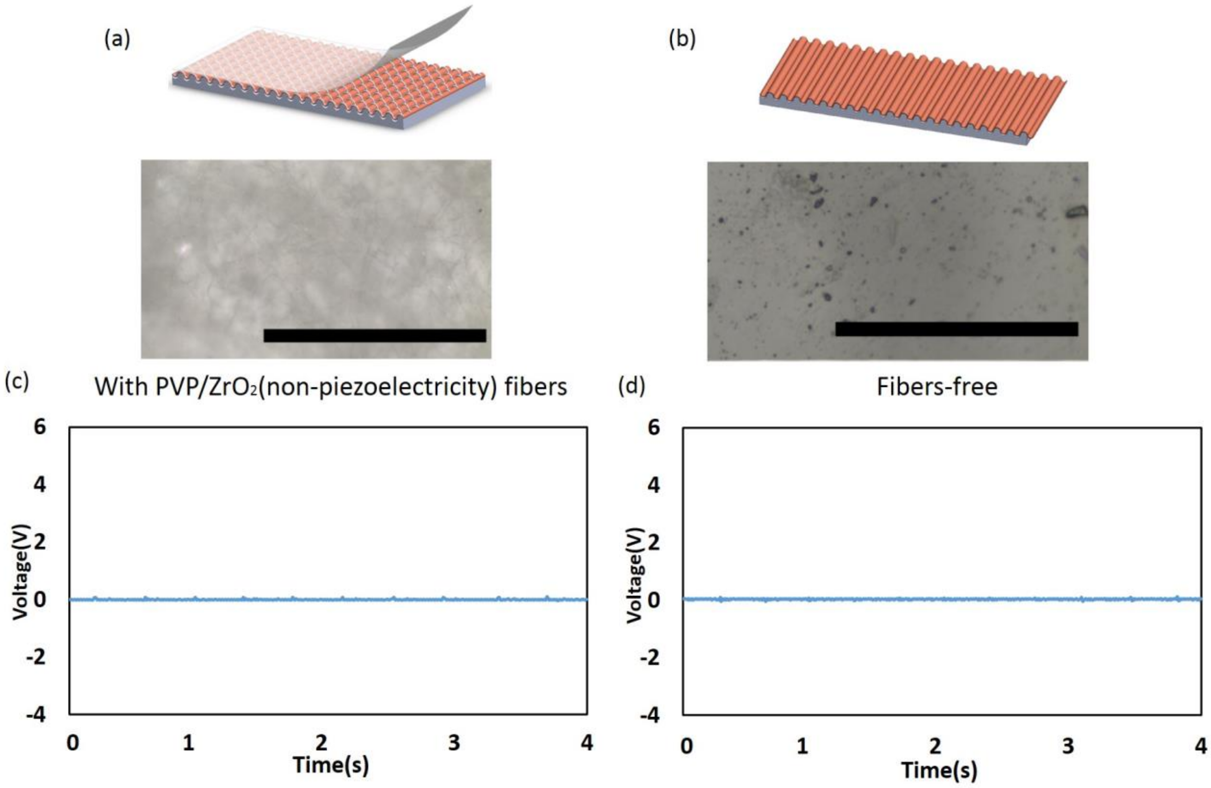

Figure S6. Output voltages of (a) non-piezoelectric fibers (PVP/ZrO2) obtained via the conventional electrospinning process (15KV, 20cm syringe-to-collector distance), (b) fibers-free substrates subject to continuous stretch and release. Respective output voltages in (c)-(d) are experimentally measured to be within the noise level of ~0.02V and below. All data are measured when the two WSSs are operated at the same strain (0.5%), strain rate, and frequency (4Hz).

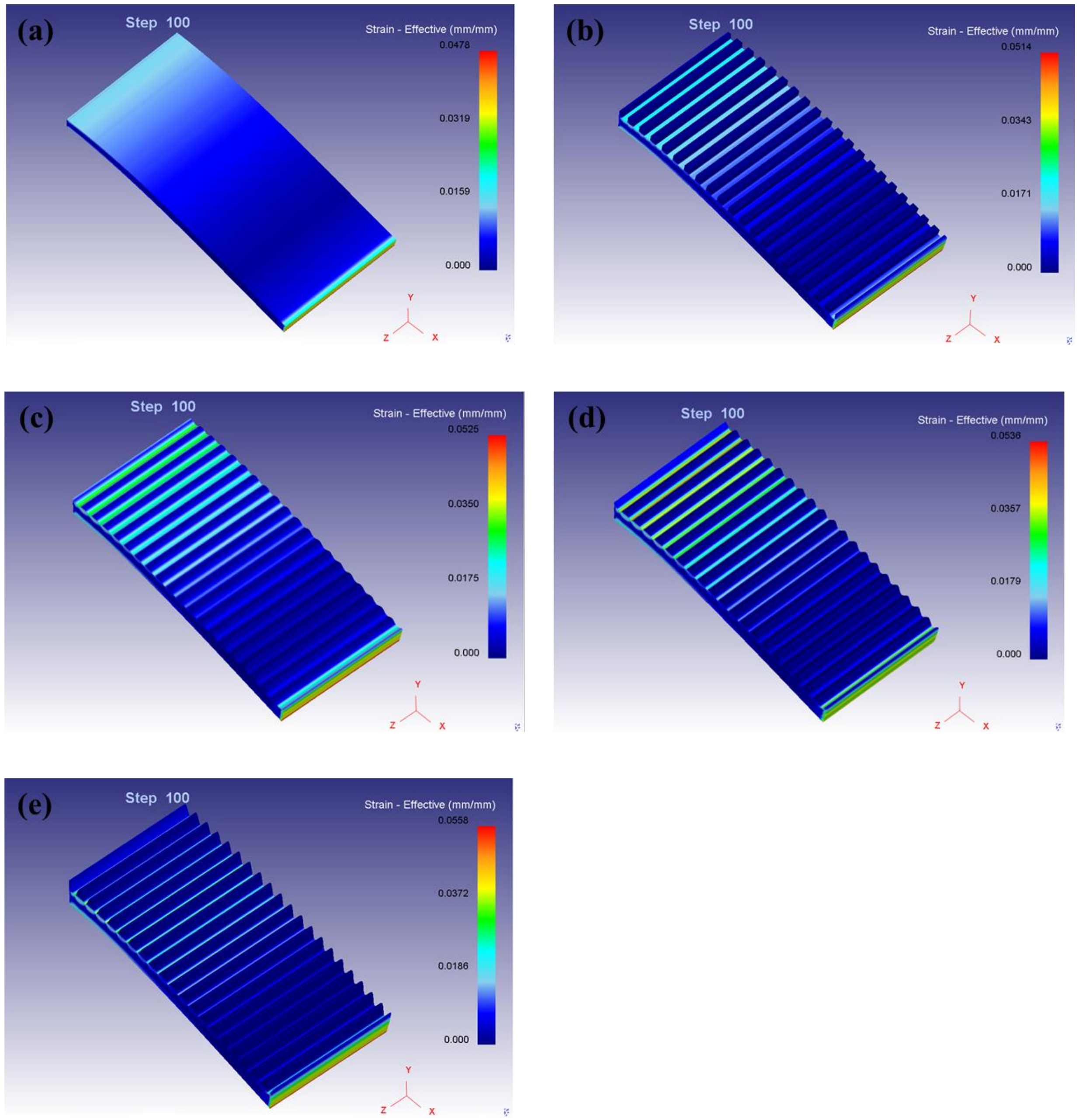

Figure S7. Based on the finite element simulation (DEFORM), the substrates experienced various strain for different topologies as follows: (a) planar surface, (b) square surface, (c-e) sinusoidal surfaces of 0.5sin(x), 1.0sin(x), 1.5sin(x), respectively. The simulation was performed of the WSS acted as the cantilever beams with one end fixed and the other end moving downwardly 8mm. As shown in the simulation results, the strain distribution of various topological surfaces exhibited different strain values. The simulated peak tensile strain with maximum strain values are 0.0478/0.0514/0.0525/0.0536/0.0558 mm/mm. As simulated in the tensile deformation process, the piezoelectric charge can be accumulated across the PVDF nanofibers across the various topologies. This phenomenon can be partially attributed to the reason why the sinusoidal wavy surface had the better electrical output. Furthermore, the higher the sinusoidal amplitude, the larger strain exerted on the substrate and thus the higher strain and

electrical output on the NMFs

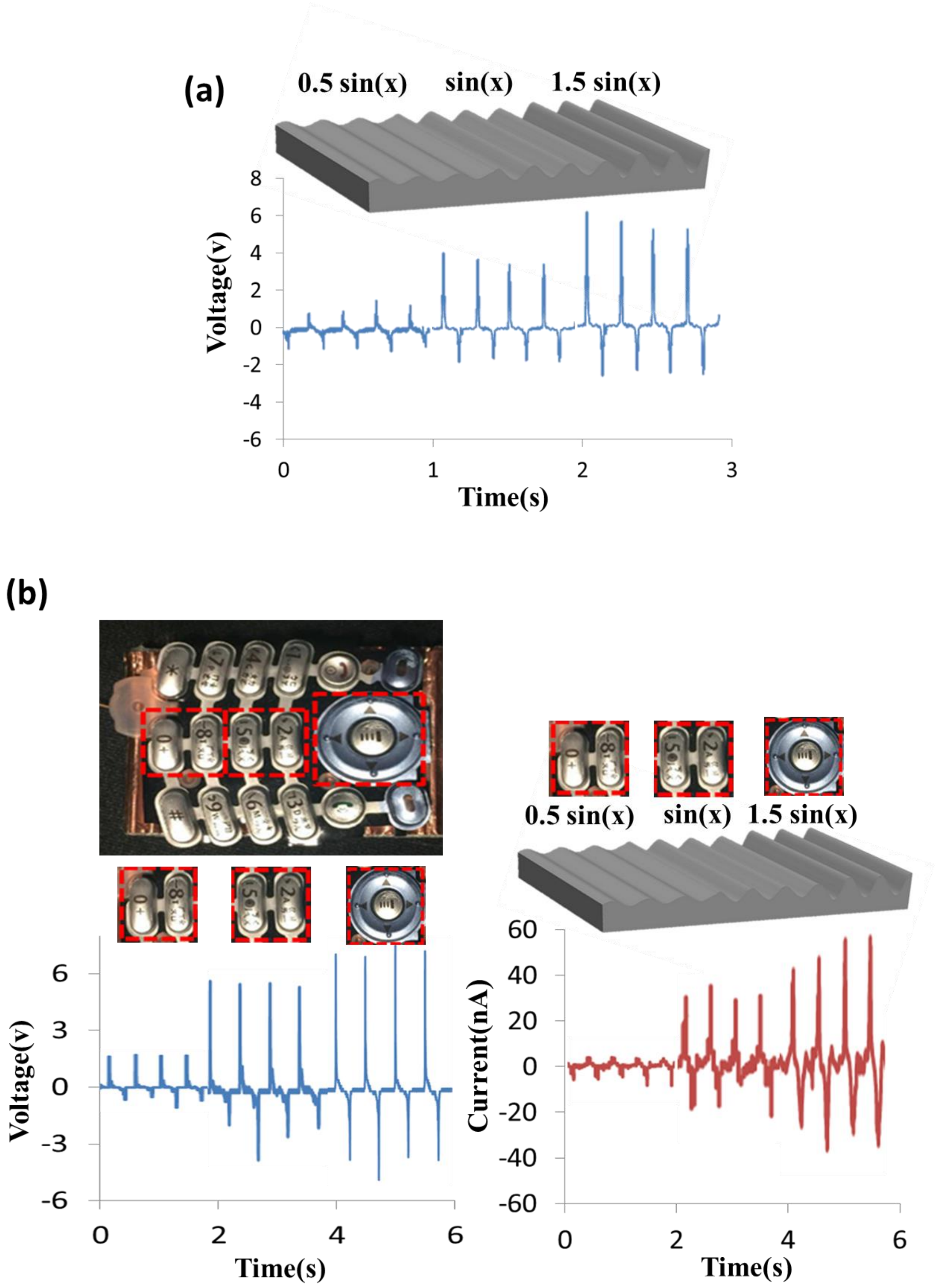

Figure S8. Combination of three different amplitude of sinusoidal wavy surfaces of A sin(x),

where the amplitude A values are 0.5, 1, 1.5mm respectively. The average output voltage are experimentally found to be proportional to the amplitude and the reason is mainly attributed to longer electrospun fibers and higher strain as experienced during the repeated press/release cycle. (b) Application of the integrated WSS device with the button of mobile phone. The average output voltages and currents generated by integrated WSS device of amplitude of 0.5/1/1.5mm height are ~ 1.5V/5V/7V and ~ 5nA/30nA/50 nA respectively in the striking motion of button movement.

(a)

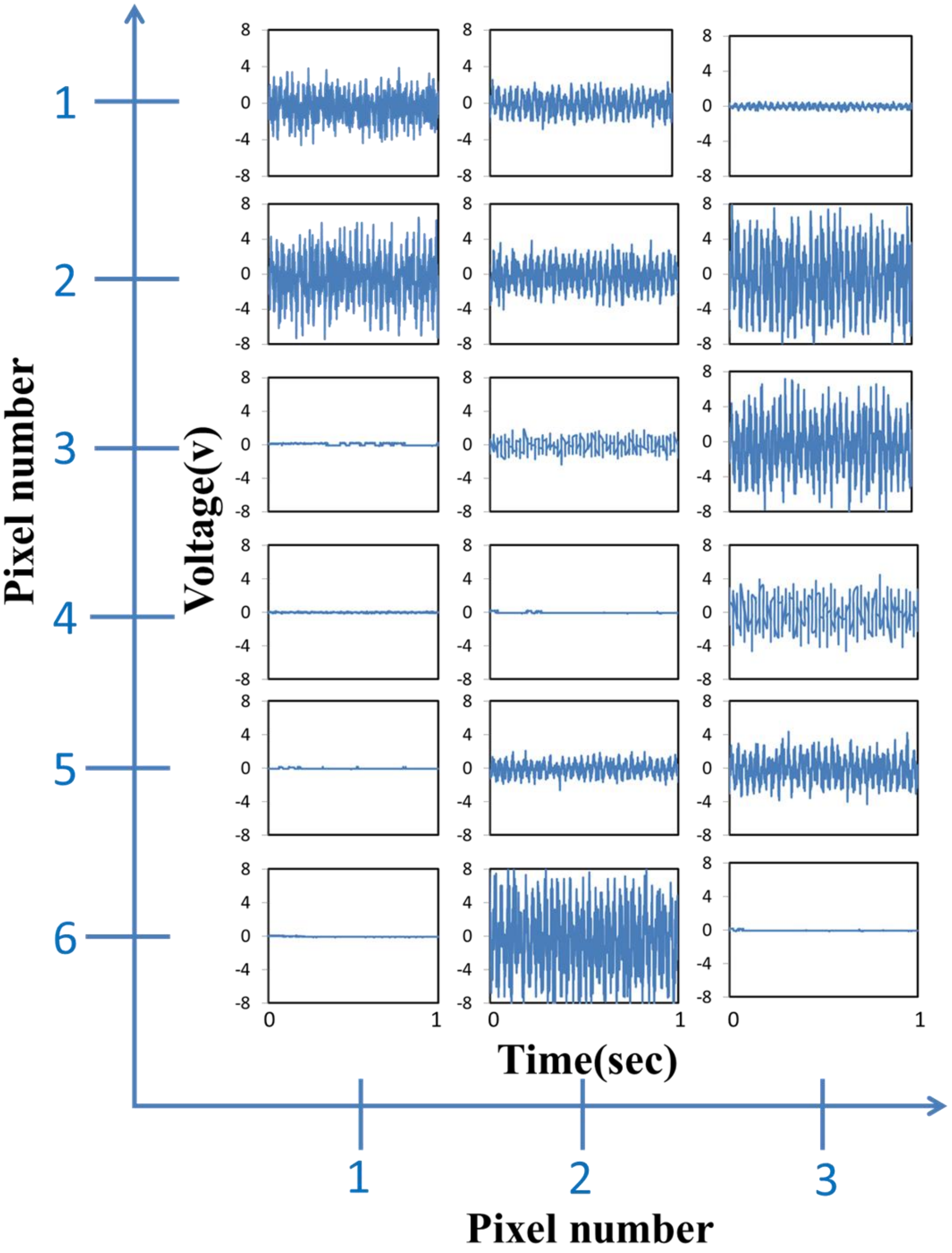

Pixel number
Voltage(v)
Time(sec)
Pixel number
1
2
3
4
5
6
8
4
0
-4
-8
0
1

(b)

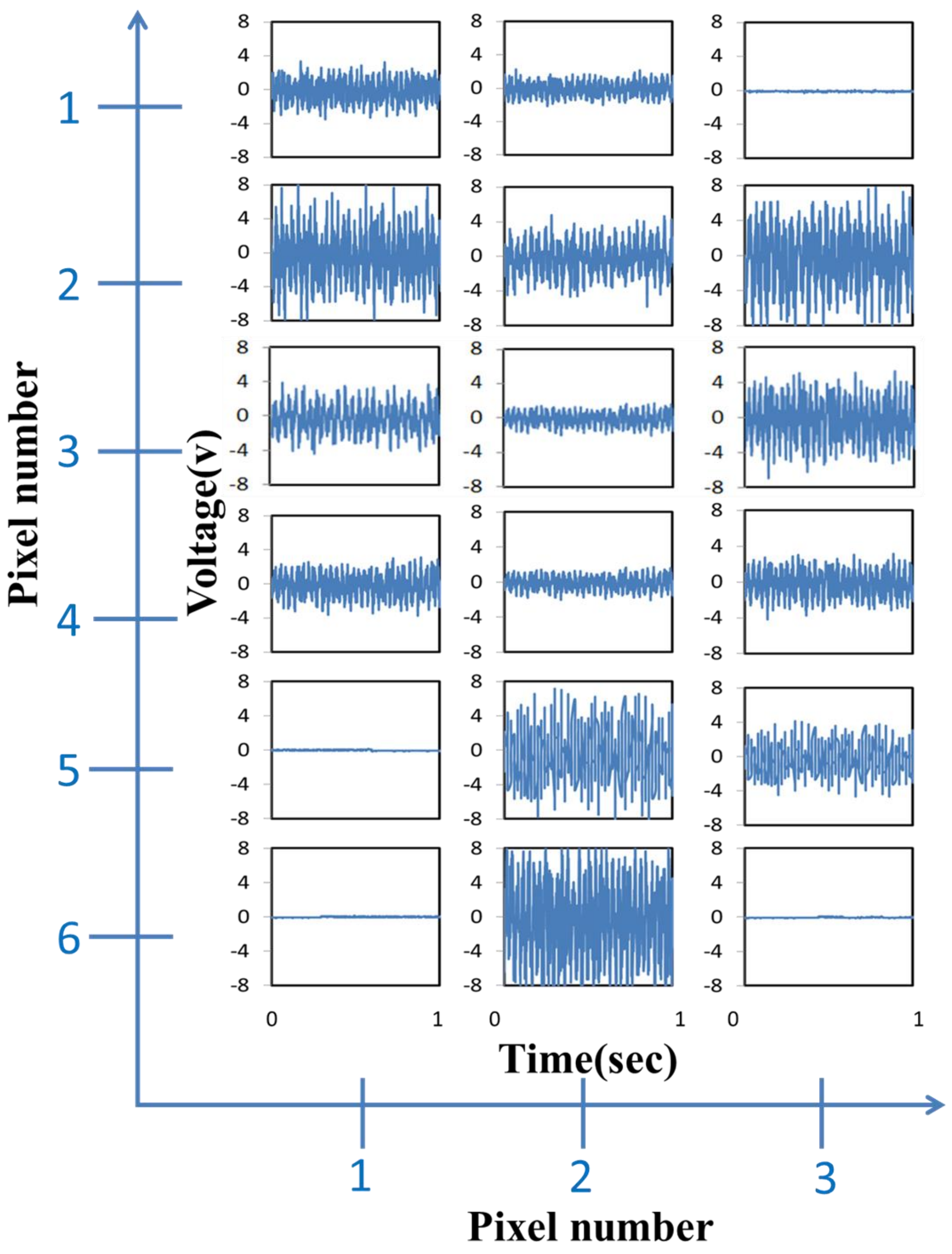
Pixel number
Voltage(v)
Time(sec)
Pixel number
1
2
3
4
5
6
8
4
0
-4
-8
0
1

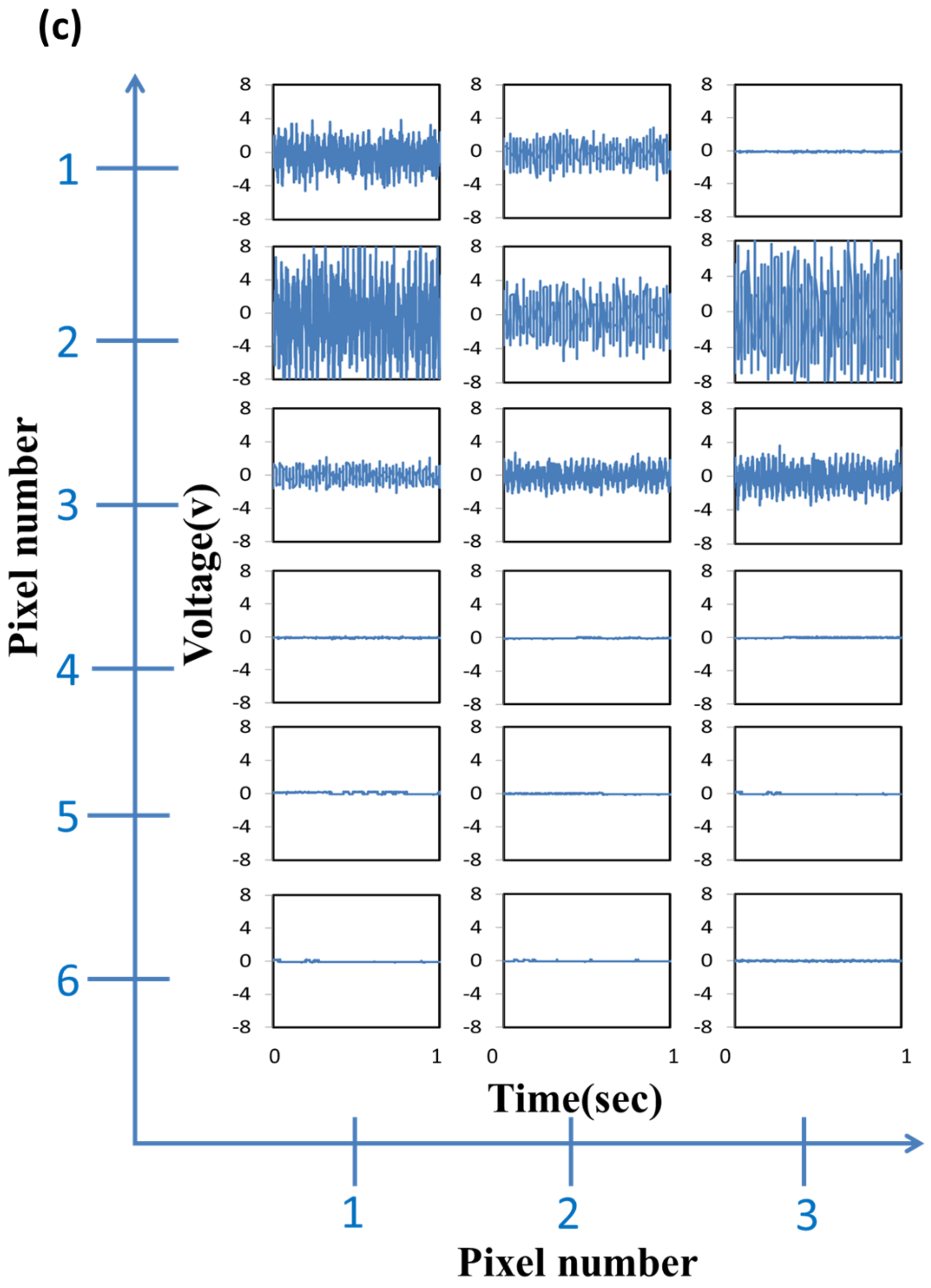

Figure S9. Raw data for open-circuit voltage for each array of WSS for detection of foot pressure. (a) subject A with normal foot, (b) subject B with flatfoot, (c) subject A with tip-toed position.

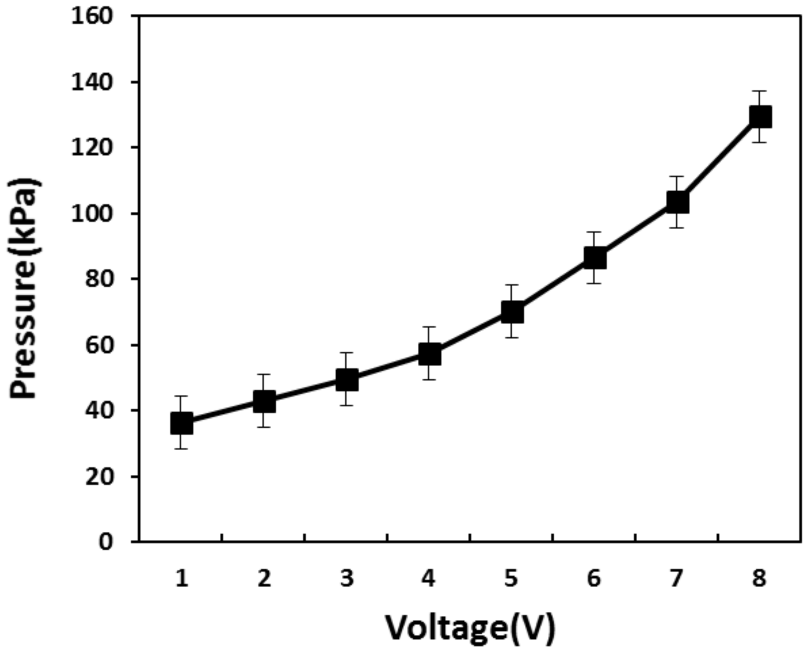

Figure S10. Characterization of the WSS performance and the relationship between the applied pressure and electrical voltage, showing the promising potential as the self-powered pressure sensor. The human motion-induced testing mode is performed by pressing on top of the device in a cyclic and reciprocating motion at the frequency of 4 Hz.
